# Proteogenomic Characterization of the Cement and Adhesive Gland of the Pelagic Gooseneck Barnacle *Lepas anatifera*

**DOI:** 10.3390/ijms22073370

**Published:** 2021-03-25

**Authors:** Dany Domínguez-Pérez, Daniela Almeida, Josef Wissing, André M. Machado, Lothar Jänsch, Agostinho Antunes, Luís Filipe Castro, Vitor Vasconcelos, Alexandre Campos, Isabel Cunha

**Affiliations:** 1 CIIMAR—Interdisciplinary Centre of Marine and Environmental Research, University of Porto, Rua General Norton de Matos s/n, Terminal de Cruzeiros do Porto de Leixões, 4450-208 Matosinhos, Portugal; dany.perez@ciimar.up.pt (D.D.-P.); danielaalmeida23@gmail.com (D.A.); andre.machado@ciimar.up.pt (A.M.M.); aantunes@ciimar.up.pt (A.A.); filipe.castro@ciimar.up.pt (L.F.C.); vmvascon@fc.up.pt (V.V.); amoclclix@gmail.com (A.C.); 2 Cellular Proteomics Research, Helmholtz Centre for Infection Research, Inhoffenstraße. 7, 38124 Braunschweig, Germany; Josef.Wissing@helmholtz-hzi.de (J.W.); Lothar.Jaensch@Helmholtz-HZI.de (L.J.); 3 Biology Department, Faculty of Sciences, University of Porto, Rua do Campo Alegre, s/n, 4169-007 Porto, Portugal

**Keywords:** underwater adhesion, cement protein, shotgun proteomics, iBAQ, protein expression, mass spectrometry

## Abstract

We focus on the stalked goose barnacle *L. anatifera* adhesive system, an opportunistic less selective species for the substrate, found attached to a variety of floating objects at seas. Adhesion is an adaptative character in barnacles, ensuring adequate positioning in the habitat for feeding and reproduction. The protein composition of the cement multicomplex and adhesive gland was quantitatively studied using shotgun proteomic analysis. Overall, 11,795 peptide sequences were identified in the gland and 2206 in the cement, clustered in 1689 and 217 proteinGroups, respectively. Cement specific adhesive proteins (CPs), proteases, protease inhibitors, cuticular and structural proteins, chemical cues, and many unannotated proteins were found, among others. In the cement, CPs were the most abundant (80.5%), being the bulk proteins CP100k and -52k the most expressed of all, and CP43k-like the most expressed interfacial protein. Unannotated proteins comprised 4.7% of the cement proteome, ranking several of them among the most highly expressed. Eight of these proteins showed similar physicochemical properties and amino acid composition to known CPs and classified through Principal Components Analysis (PCA) as new CPs. The importance of PCA on the identification of unannotated non-conserved adhesive proteins, whose selective pressure is on their relative amino acid abundance, was demonstrated.

## 1. Introduction

Goose barnacles are filter-feeding marine crustaceans that live attached by the stalk to a fixed hard substrate, or to floating objects, by means of an adhesive secretion. The adhesive secretion is produced at the cement gland located at the top of the peduncle core, beneath the capitulum [1,2]. The adhesive secretion is conducted to the peduncle’s base through ducts, where it is released, allowing to holdfast the specimens in an adequate position to meet the parameter needed for their survival under a variety of hydrologic regimes according to the species ecology (i.e., in oceanic or coastal habitats, submersed or intermittently immersed in intertidal zones, in protected overhangs, crevices, in the deep sea, or directly exposed to strong waves) [3,4]. Indeed, barnacles’ cement multicomplex has been evolutionarily optimized to attach the base firmly to wet substrates, conferring plasticity and resistance, which has been inspiring for industrial applications (e.g., antifouling strategies, induction on demand of larvae settlement and fixation for aquaculture, underwater industrial and surgical glues, dental composites, biocompatible scaffolds, and coatings) that need adhesion under humid or wet conditions [5,6,7,8,9].

In general, the cement multicomplex is mainly composed by two type of proteins, one forming the core of the secretion, and another the interfacial layer [10]. The core of the secretion is composed by cohesive hydrophobic proteins named “bulk proteins”, since they are the most abundant, while interfacial proteins are less abundant hydrophilic proteins making the outside boundary of the cement complex. Interfacial proteins are essential in providing surface coupling with exterior surfaces, through non-covalent interactions, and also interaction with the interior hydrophobic bulk proteins [10]. Six barnacle specific cement proteins (CPs) have been identified to now, two bulk proteins, CP52k and -100k, and four interfacial proteins, CP19k, -20k, -43k, and -68k [11], where the numbers correspond to the approximate molecular weight in kilo Daltons. However, in our opinion, this classification has various drawbacks, since it does not provide accurate information of the CPs diversity, nor a proper definition of CPs family. Moreover, the more CPs are identified, featuring different molecular weights, the bigger the confusion for naming the CPs and CP families, as naming is based on weight, and particularly since high throughput proteomics has been applied to the analysis of cirripede adhesives, several new proteins with an enormous variety of molecular weights have been described [12,13,14,15,16,17]. CPs may have a quite different weight, while featuring very similar physicochemical properties and amino acid relative abundance, which causes their clustering in principal components analyses [17]. In some cases (e.g., CP19k and CP20k; surface coupling proteins) they may have similar weight and feature very different physicochemical properties and amino acid relative abundance. In *Pollicipes pollicipes* several putative CPs were recently reported in the cement, with a variety of molecular weights [15]. Intriguingly these barnacle CPs did not show any homology with other marine adhesive proteins [11,12], or other proteins at all, possessing no identifiable protein domains, being in some cases structurally very disordered, and that could not be named in the previously defined CP’s nomenclature. This puts a limitation to name the new CPs identified, specifically the interfacial ones, which seems to be more diverse [15]. Despite the updating of the general model of barnacle adhesion in the last years [16,18,19], the current CPs classification is becoming obsolete with the discover of new CPs [15]. Barnacles’ adhesive secretion mechanism remains misunderstood, and CPs misclassified.

**Figure 1:**
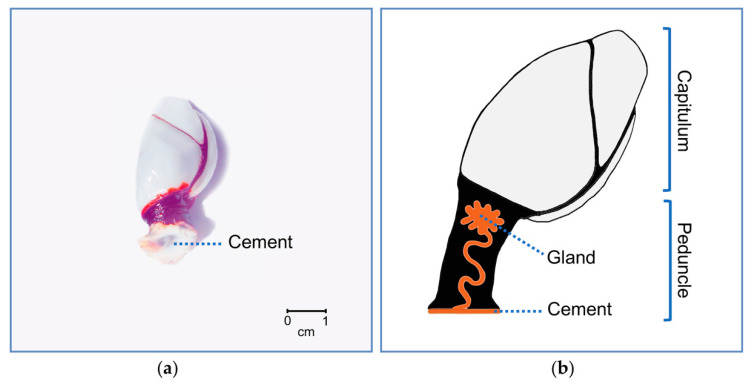
The pelagic gooseneck barnacle *Lepas anatifera* (**a**) detached from the substrate, showing the cement at the base of the peduncle; (**b**) schematic drawing showing a section of the peduncle, highlighting the cement apparatus, formed by clusters of adhesive-secreting cells that together constitute the gland, located in the upper central core, embedded in an apical layer of connective tissue, just beneath the capitulum. Gland secretion passes through a network of ducts that carry the adhesive to the base of the peduncle forming the cement.

Indeed, CPs remain underexplored, also in free living pelagic species, such as in the common goose barnacle, *L. anatifera*, most abundant in tropical and subtropical waters, where sea temperatures exceed 18–20 °C [20]. This species is of particular interest since specimens can be found attached to a variety of floating objects, including driftwood, bottles, boats, buoys, macroalgal rafts, and turtles [20,21], and eventually in fixed rocky substrates (Figure 1a), which foresees it might have a very plastic adhesive. They need to cluster in turfs close enough to mate to conspecifics, since despite being hermaphroditic, require cross-fertilization, and fecundation is internal [22], which makes strong holdfast a vital characteristic. Moreover, in the case of *P. pollicipes*, they live in the most unprotected frontal rocks of the shores, or in narrow crevices, where hydrodynamic forces are enormous, to feed on zooplankton that wave carry [23], which further accentuates this vital character of adhesion. For these species detaching means not mating and not eating. However, the properties of their adhesive secretion still need to be further characterized, considering the characteristics and abundance of CPs, as well as their relationship with substrate characteristics. Notwithstanding, *L. anatifera* is an interesting species to explore this relation, because of its substrate preference diversity, in opposition to other stalked barnacle species which have stricter substrate preferences [24].

The cement apparatus of the pelagic barnacle *L. anatifera* (Figure 1b) is found at the top of the peduncle’s core, formed of a single type of adhesive-secreting unicellular gland clusters, mostly just below the mantle cavity, and a network of ducts that coalesce and carry the adhesive to the base of the peduncle [1,2,25]. In ripen individuals, some adhesive secreting cells are intermingled with the ovary, but most of them are located between the ovary and the capitulum. The presence of large nucleoli in the nucleus and the large amounts of rough endoplasmic reticulum in the cytoplasm of these cells suggest an intense protein synthesis. The cytoplasm of the adhesive-secreting cells also features numerous small electron-dense secretory vesicles, which stained positively for proteins (tetrazonium), polysaccharides (PAS), but not for the presence of lipids (Sudan black), in histological studies [1]. Contrarily, on barnacles’ cyprids, the adhesive is reported to be a bi-phasic system containing lipids and phosphoproteins, the two distinct phases contained in two different kind of granule, at cyprid cement gland cells [26]. Furthermore, post-translational modifications do not seem to play a role in adult barnacles’ adhesion, except for the glycosylation of MR52k [27], which is in line with the positive PAS dying observed of the electron-dense secretory vesicles in the gland cells cytoplasm.

The combination of transcriptomic and proteomic approaches has resulted in a powerful tool for a high throughput discovery of barnacle’s CPs [15]. Thus, this study was focused on the proteogenomic characterization of *L. anatifera* (Figure 1) adhesion system. The protein composition of *L. anatifera* cement multicomplex and cement gland was quantitatively studied using label-free LC-MS proteomic analysis combined with bioinformatics approaches for protein identification and classification. The proteogenomic analyses applied allowed to identify the known CPs both in the proteome and gland transcriptome, as well as a group of unannotated proteins. As previously described in *P. pollicipes*, some unannotated proteins identified in the cement proteome of *L. anatifera* were abundant. The principal component analyses revealed some of them as new CPs, since after PCA they grouped with clusters of canonical CPs previously described, either bulk or surface coupling proteins, due to the selective pressure for conservation of relative amino acids composition in each CP group [15]. Moreover, these proteins lacked annotation and/or conserved domains, sharing some physico-chemical features with CPs, e.g., molecular weight, isoelectric point, hydrophobicity, amino acid relative composition, secondary structure composition, and protein disorderliness [15,16,17]. This finding allowed to conclude that some unannotated proteins identified here, as well as those previously discovered in *P. pollicipes*, are indeed new canonical CPs, and some possibly belonging to other CPs families not yet defined, as they are very abundant in the adhesive, but did not cluster with the canonical groups characterized so far. Our findings reaffirm the limited knowledge we have on barnacles’ CPs diversity, as well as the urgent need for a functional nomenclature for barnacles CPs, to replace the existing one based on their molecular weight.

## 2. Results and Discussion

### 2.1. Protein Identification

**Figure 2:**
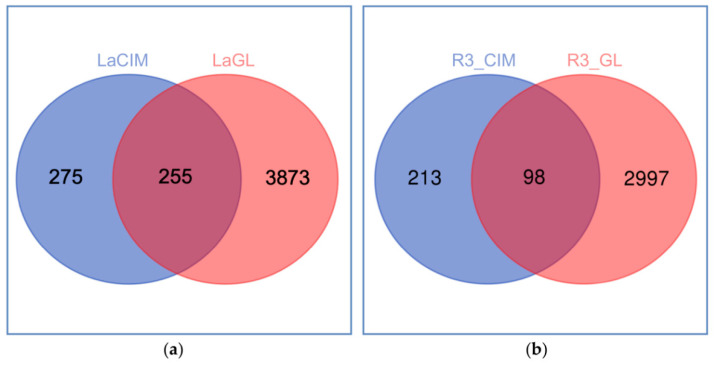
Venn diagram of the proteins identified with MaxQuant freeware in the cement and gland proteome of pelagic gooseneck barnacle *L. anatifera*. Unique and shared proteins between (**a**) all proteins identified in the cement (LaCIM) and gland proteome (LaGL); and (**b**) all proteins identified in the three biological replicates of the cement (R3_CIM) and gland proteome (R3_GL).

The shotgun proteomic approach employed to profile the proteome of the cement gland and secreted cement itself, of the pelagic gooseneck barnacle *L. anatifera* (Figure 1), allowed to identify 11,795 peptide sequences in the gland (Table S1) and 2026 peptide sequences in the cement (Table S2). After filtering (contaminants, “only identified by site” and REV_ removal), a total of 4128 proteins clustered in 1689 proteinGroups prevailed in the gland proteome (Table S3), and 530 proteins clustered in 217 proteinGroups in the cement (Table S4). Altogether, 4403 unique proteins were identified, 255 of which shared by the gland and cement, comprising nearly 50% of the total unique proteins (Figure 2a), as previously described for *P. pollicipes* [13]. Of the 3308 proteins identified in the three replicates, 3095 and 311 were found of the gland and cement samples, accounting for 1219 and 132 proteinGroups, respectively, whilst only 98 proteins overlapped all replicates analyzed, accounting for only 31.5% (Figure 2b; Tables S5 and S6), a much smaller relative amount than when all proteins are considered (48%). Among the proteins identified in the three samples, 3308 were non-redundant, being 2997 unique proteins in the gland proteome and 213 exclusive of the cement (Figure 2b). The original MaxQuant output files containing all proteins identified (proteinGroups), without filtering, can be found at Table S7 for the gland and at Table S8 for the cement.

### 2.2. Quantitative Proteomic Analyses

**Figure 3:**
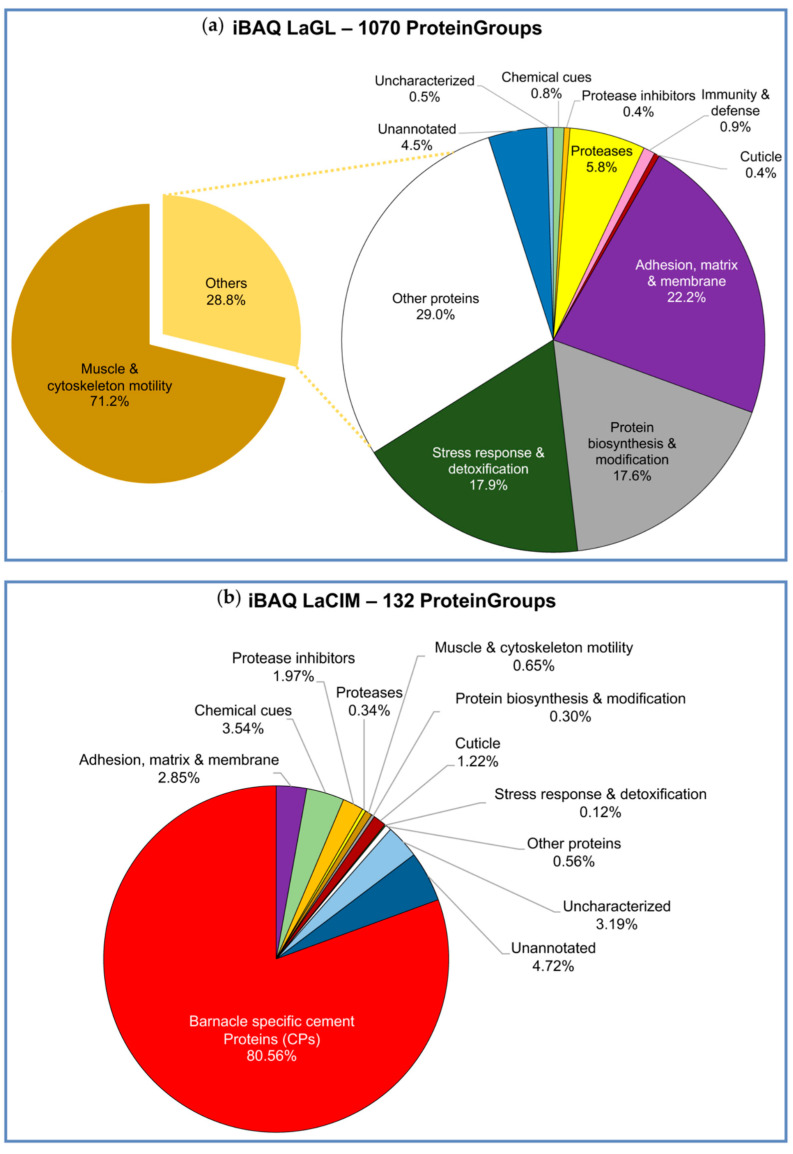
Global protein composition by functional groups in (**a**) the gland proteome (LaGL); and (**b**) cement proteome (LaCIM) of the goose barnacle *Lepas anatifera*. The proportion of functional groups was based on the absolute protein abundance using intensity Based Absolute Quantification (iBAQ) score calculated by MaxQuant. Only those proteins found in the three biological replicates (three valid values) of the two studied samples were selected. In total, 1070 proteinGroups were used in the analysis of the gland (LaGL), and 132 in the cement (LaCIM).

**Figure 4:**
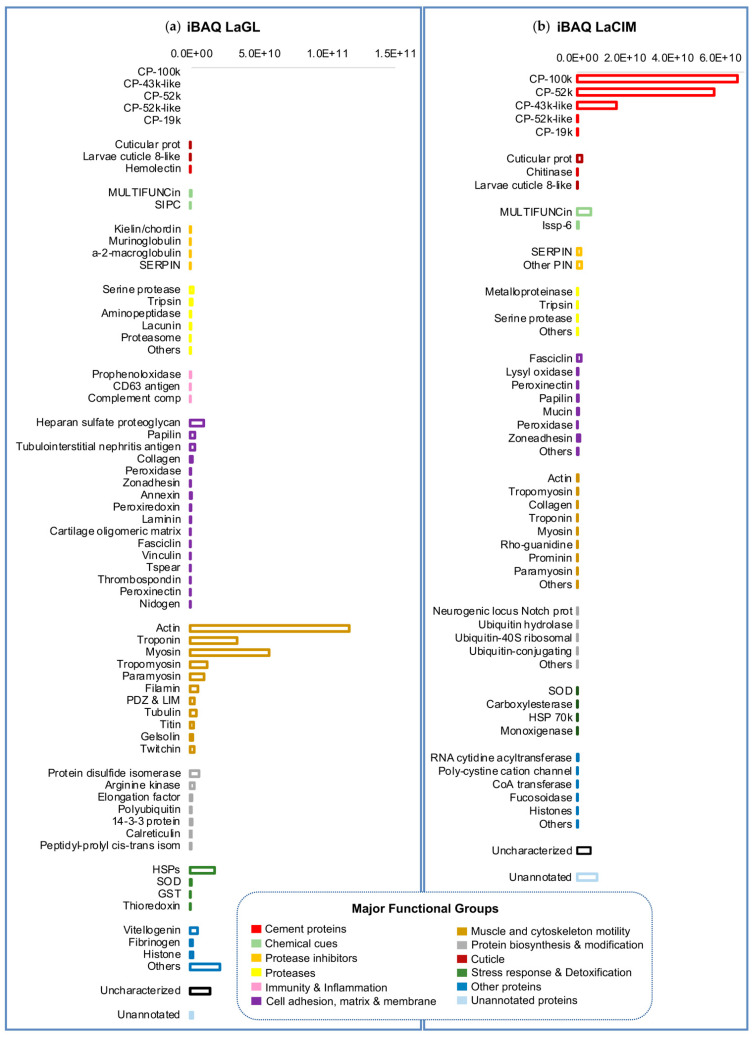
Relative protein abundance within (**a**) the gland proteome (LaGL); and (**b**) cement proteome (LaCIM) of the cosmopolitan goose barnacle *Lepas anatifera*. Absolute protein abundance of broad protein families, shown as a single bar, was obtained using an intensity Based Absolute Quantification (iBAQ) score calculated by MaxQuant. The horizontal scales of bars differ between samples (LaGL and LaCIM), being highest in the gland proteome.

Protein expression in the gland and cement was determined as absolute protein abundance using an intensity Based Absolute Quantification (iBAQ) score calculated by MaxQuant (Tables S5 and S6, respectively). Both gland and cement proteome, showed a similar profile to other barnacles, mainly to the *P. pollicipes* proteome, which were studied using the same methodology [15]. Likewise, the composition of the *L. anatifera* cement gland was dominated by proteins involved in muscle and cytoskeleton motility, accounting for 71.2% (Figure 3a). The majority corresponded to actin, myosin, troponin, and tropomyosin, including other contractile and structural proteins (Figure 4a). In addition, proteins involved in “adhesion, extracellular matrix and membrane” corresponded approximately to 6.4% of total expression (Figure 3a), being heparan sulphate proteoglycan, papilin, and collagen the most expressed within this functional group (Figure 4a). The group of proteins involved in “protein synthesis and modification” accounted for 5.1% of total expression, similarly to group of “stress response and detoxification proteins” (5.2%), mostly constituted by heat shock proteins (HSPs) that had an important representation. Proteases (1.7%) where also quite well represented, being serine proteases and trypsin the most expressed. The group of proteins that remained uncharacterized or unannotated accounted for 1.4% of total expression (Figure 3a). Minor components such as chemical cues, proteinase inhibitors, immune and defense and, cuticle proteins were also detected in the cement gland.

The canonical barnacle’s cement proteins were not detected in the quantitative analyses of the cement gland at the proteomic level (Figure 4a), but some bulk proteins such as CP100k (ATB53757.1; AGS19349.1) and CP52k (ATB53756.1) were found at relatively high expression in the gland transcriptome (Table S9). Not very differently, in *P. pollicipes* only CP100k was detected through the proteogenomic analyses performed and at a very low expression level [15]. The absence of the canonical CPs in the gland at proteomic level and the relative high abundance of its encoding transcript, could be related with the sensitivity of the methodology and the relative abundances. In addition, the translation to proteins in the gland could be further lowered than transcription, once the barnacles are established and fixed to the substrate, being the production of some CPs reduced or down-modulated in the gland, both in pelagic species and species inhabiting rocky intertidal systems [28]. Moreover, it has been demonstrated that the synthesis of the permanent adhesives only occurs during the early cyprid stage [16,29]. However, a low level of protein production must always be necessary to repair eventual detachment due to hydrodynamism, and to provide for displacement to occur. Indeed, barnacles in development can periodically secrete primary cement to achieve firm attachment [30], but once adhered, in many species adult barnacles can neither move freely on the surface nor actively detach from the substrate [16]. Relocation of adult *P. pollicipes* along the substrate after settlement, but mainly by juvenile along the stalk, was confirmed by Kugele and Yule [30], and also in acorn barnacles by Moriarty and coauthors [31]. Contrarily, *L. anatifera* is unable to relocate voluntarily; no evidence of relocation of animals from the capitulum to the substratum, or base of host animals was lacking [30], which may explain such extreme down-regulation of CPs production at the gland in this species.

**Figure 5:**
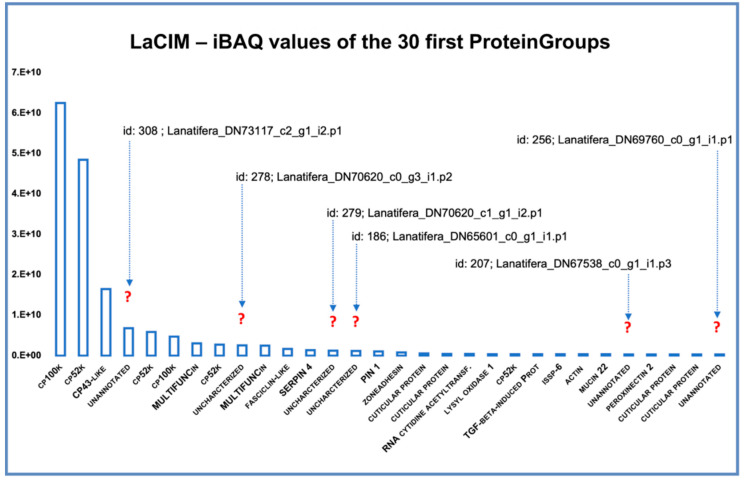
The 30 most expressed proteins in the cement proteome (PPCIM) of the cosmopolitan gooseneck barnacle *Lepas antifera*, based on the absolute protein abundance. The expression of the 30 most expressed proteins (X-axis) is represented by the intensity Based Absolute Quantification (iBAQ) score (Y-axis) calculated by MaxQuant. Question marks indicate six highly expressed proteins with no homology (named as unannotated in X-axis), found in the cement proteome, their protein Group id, and the name of the corresponding leading protein.

On the contrary, cement proteome was dominated by barnacle’s cement canonical proteins (CPs), and in minor amount by unannotated and uncharacterized proteins, chemical cues, protease inhibitors and adhesion, matrix, and membrane proteins (Figure 3b). Among canonical proteins, bulk proteins CP100k and then CP52k were the most expressed, contrarily to *P. pollicipes*, in which the most expressed bulk CP was CP52k, and only then CP100k [15]. Regarding surface coupling proteins, CP43k was the most expressed in *L. anatifera*, followed by CP19k with less expression. By contrast, *P. pollicipes* showed CP19k as the most expressed surface coupling protein, while the CP43k was not even represented in the proteome [15]. Unannotated and uncharacterized proteins accounted for 7.9% of total proteins (Figure 3b). It should be noted that six proteinGroups classified as unannotated or uncharacterized were listed among the 30 most represented proteins on the cement (Figure 5). Due to their high abundance in the cement, we suspected that these proteins might be functional adhesive proteins belonging to previously characterized canonical CP families or to other families never detected or characterized before, either bulk or interfacial proteins, or having even a different function/location from those previously described.

As discussed above, holdfast is essential for the survival of cirripedes, provided by the cement secretion, whose properties, for their importance to survival, must be evolutionary selected according to the species ecology. Herein, the CPs composition of *L. anatifera* is described for the first time, a cosmopolitan species found in a variety of floating substrata adrift in the ocean, or fixed but swinging or slightly moving with currents, in conclusion, a little selective species for the fixation substrate.

**Figure 6:**
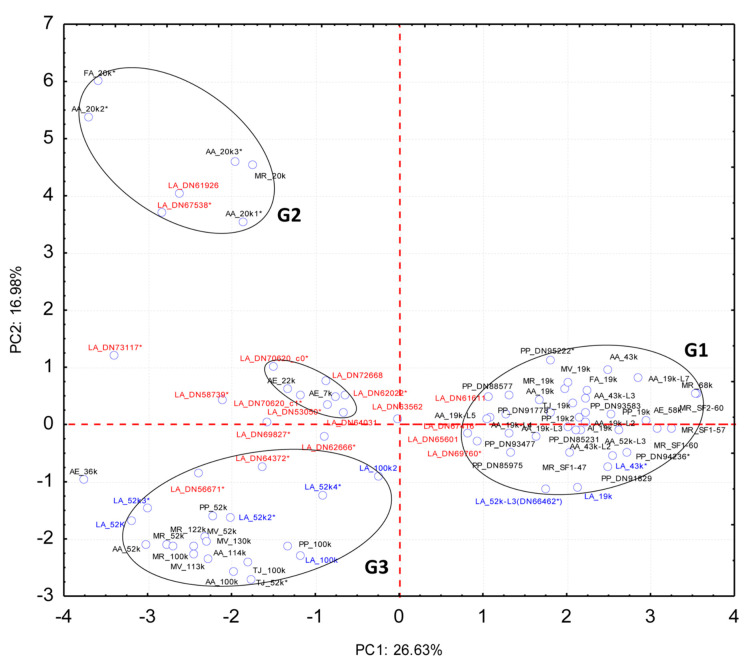
Principal component analysis based on the amino acid relative composition of proteins, considering 53 previously characterized cement proteins, including the 8 annotated *L. anatifera* annotated identified during the present study through BLAST, and those 20 unannotated proteins identified during the present study, which was not possible to annotate through BLAST or conserved domains. The protein groups previously defined, correspond to surface couple proteins CP19k, -43k, -58k, and -68k (G1), CP20k (G2), and bulk proteins CP52k and -100k (G3) [17]. The two principal components (PC) extracted explain 43.61% of the total variability of data. Blue—Automatically annotated *Lepas anatifera* proteins; Red—unannotated *L. anatifera* sequences; Black—cement proteins (CPs) previously characterized, available at NCBI or retrieved from the bibliography. Asterisk marked sequences are incomplete.

Nine CPs were identified by homology to the canonical CPs of other species (DN69987_c0_g1_i2.p1, ATB53757.1: CP100k (*P. pollicipes*); DN63945_c0_g1_i4.p1, ATB53756.1: CP52k (*P. pollicipes*); DN40455_c0_g1_i1.p1, AQA26377.1: CP43-like3 (*Amphibalanus amphitrite*); DN67731_c0_g1_i1.p1, ATB53756.1—CP52k (*P. pollicipes*); DN69987_c0_g2_i1.p1, AGS19349.1: CP100k (*A. amphitrite*); DN63945_c0_g1_i2.p1, ATB53756.1: CP52k (*P. pollicipes*); DN62610_c0_g1_i4.p1, ATB53756.1: CP52k (*P. pollicipes*); DN66462_c0_g1_i1.p1; AQA26375.1: CP52k-like3 (*A. amphitrite*); DN68159_c0_g1_i3.p1, ATB53755.1: CP19k (*P. pollicipes*)) (Table 1 and Table S8). The protein DN66462_c0_g1_i1.p1, the 31st most expressed protein, was automatically annotated in the cement proteome as CP52k-like3 (Table 1 and Table S8), but according to the PCA analysis performed on its amino acid composition, this is not a bulk protein since it clusters with G1 proteins (Figure 6), being otherwise a surface couple protein. This protein is encoded by the transcript Lanatifera_DN66462_c0_g1_i1 (see Table S9) with accession GGJN01121414.1 in the Transcriptome Shotgun Assembly (TSA) Database, corresponding to the biosample SAMN08662092, bioproject PRJNA437397. The other cement proteins detected, CP100k, -52k, -43k and -19k, were homologous to those previously found, ATB53757.1, ATB53756.1, AQA26377.1, and ATB53755.1, respectively [17].

In addition, other abundant proteins in the cement proteome were associated with “chemical cues” (Figure 3b), among them MULTIFUNCin and issp-6 were the most represented (Figure 4b and Figure 5). MULTIFUNCin is a multifunctional glycoprotein cue previously found in another barnacle’s cement [15,18,32]. This glycoprotein is seemingly involved in habitat selection (settlement) by conspecific barnacle larvae, adhesion and defense [32]. On the other hand, issp-6 (S10) is a protein member of hemolymph juvenile hormone binding (IPR010562) family of proteins, [15]. This protein family is related to the juvenile hormone pathway, which is mainly involved in metamorphoses and development in cyprids [33]. Settlement inducing protein complex proteins (SIPC) are glycoproteic chemical cues that were found to be very abundant in the rocky shore goose barnacle adhesive, where they represent 3.2% of total proteins [15], but not found in the adhesive of ocean drifting species, despite being present in the gland. In replacement, *L. anatifera* features issp-6 in its cement multicomplex (Figure 4). Despite both species being gregarious, chemical cues are much more represented in *P. pollicipes* adhesive (12.1%), than in *L. anatifera* one (3.5%), possibly related to the strategy of each species to thrive, one moves in the ocean to meet the patches of larvae ready to settle, the other is sessile and needs to attract larvae to settle over, by means of producing larger amounts of chemical cues.

In addition, proteins involved in “adhesion, matrix, and membrane” and “protease inhibitors” were also relatively abundant in the cement, with approximately 2.9% and 2% of relative abundance, respectively (Figure 3b). The enzyme lysyl oxidase was among the 30 most expressed proteins (Figure 5). This enzyme was also abundant in *P. pollicipes* cement proteome [15], and their active role in attachment demonstrated through proteomic and enzymatic approaches in in the adhesive layer of adult *Amphibalanus amphitrite* barnacles [18]. Lysyl oxidase was assigned to the modification of cement components, likely involved in lysine/arginine protein cross-linking, but also in collagen’s and elastin fibrils’ cross-linking [18].

Other components were related to “protease inhibitors” (2%), “cuticle” (1.2%), and minor components “muscle and cytoskeleton motility”, “protein biosynthesis and modification”, and “stress response detoxification”, in this order (Figure 3b), whereas some proteins found in the quantitative analyses remained with unknown function, “unannotated” and “uncharacterized”, which will be discussed below.

#### Unannotated Proteins of the Cement Proteome

Proteins without annotation, uncharacterized, or just predicted, were found to be abundant—8.0%—in the cement proteome (Figure 3b, Figure 4b, and Figure 5). Some of those proteins were also found in the gland at proteomic (Figure 3a and Figure 4a, Table S5) and transcriptomic level (Table S9). To figure out the biological function of such proteins, some additional analyses were performed. A total of 132 proteinGroups were blasted against the Non-Redundant protein database (nr at NCBI) using automatic adjustment of the BLASTp program. Of all, a total of 19 proteinGroups remained unannotated, without any protein homology description, or known conserved domains (Table S10). The results of these analyses were also included in the figures previously shown, and detailed information of Blast search and protein sequences can be found in Table S10.

Afterwards, a Principal Component Analyses (PCA) was conducted on the unannotated and uncharacterized proteins, together with known cement adhesive proteins of various species (Figure 6; Table S11) to observe clustering. PCA used the relative residue composition (%) of 19 barnacle specific cement proteins obtained in the present study in *L. anatifera* cement proteome, which were classified under “unannotated” and “uncharacterized” proteins, to compare to 53 previously identified, classified and characterized cement specific proteins of various barnacle species, gathered from NCBI and literature, belonging to 8 different barnacle species (*P. pollicipes*, *A. amphitrite*, *A. improvisus*, *A. eburneus*, *Fistulobalanus albicostatum*, *Megabalanus rosa*, *M. volcano*, and *Tetraclita japonica*). The analysis allowed to observe the clustering patterns of the unannotated and uncharacterized proteins, with the groups of proteins previously defined [17] (Figure 6). The two first principal components (PC1 and PC2) extracted by the PCA explained 43.61% of the total data variation (26.63% and 16.98%, respectively), allowing to observe proteins grouping as a function of the relative amino acid composition (Figure 6). PC1 discriminated G1 from the other two groups, while PC2 allowed for the separation of CP20k (G2) from the other two groups (G1 and G3). Regarding three CPs of *A. eburneus*, CP36k, -22k, and -7k identified by Naldrett and Kaplan [34], one of them, AE_36k did not group with any CPs group, similarly to other 5 unannotated cement protein that did not group neither with G1, G2, nor G3.

The PCA situated four of the unannotated proteins (DN61611, DN67416, DN65601, and DN69760) in the proximity of G1 (surface coupling proteins which includes CP19k, -43k, -58k, and -68k families of cement proteins), two proteins (DN61926 and DN67538) in the vicinity of G2 (surface coupling proteins of CP20k family), and two proteins (DN56671 and DN64372) near G3 proteins (bulk proteins of the families CP52k and -100k). Of the remaining 11 proteins, 6 (DN72668, DN62022; DN64031, DN53050, DN70620_c0, and DN70620_c1) clustered with AE_22k and AE_7k forming a new 8 protein cluster, while 5 of them (DN73117, DN58739, DN69827, DN62666, and DN63562) did not cluster with any group at all. Whether the cement proteins that did not cluster with the previously defined G1, G2, and G3 groups have an adhesive function or a different function in the cement multicomplex, it is yet to be determined, since the techniques herein used do not allow to determine that. It is known that they are barnacle cement specific, because they were indeed identified in samples of cement and do not have homology to any other proteins of the non-redundant protein database at NCBI. Moreover, some of these proteins are quite abundant, as for instance the DN73117, which is the fourth most represented protein in the cement, and DN70620_c0 and DN70620_c0, which were the 9th and the 13th. This makes us suspect that at least this very represented unannotated proteins might have a function that has to do with the very function of the cement itself, which is to adhere, or else to give structure to the cement.

One of the cement proteins picked from NCBI, AA52-3L, was misclassified according to the PCA analysis performed. Based on the relative amino acid composition of this *A. amphitrite* protein, PCA situates it in G1 (Figure 6), but according to the authors, it is as a bulk protein, CP52k-like [12]. In the case of being a bulk protein, it should group with G3 proteins, instead of G1. Since this protein was used to automatically annotate the *P. pollicipes* PP_52k-L identified and annotated in a previous work [15] and the LA_52k-L3 (LA_DN66462) in the present work, this two proteins were also misclassified. The two proteins are smaller in length and lighter than CP52k proteins, and their physico-chemical properties also corroborate that they are surface coupling proteins of G1 (Table 1, Table S11, and Table S13).

Regarding other characteristics of cement proteins, these are presented on Table S11 and Table 1, the former presenting the characteristics of the 53 adhesive proteins of various barnacle species previously characterized, whose sequences were available at NCBI, and the latter, the characteristics of the 19 proteins found in *L. anatifera* cement proteome, which could not be annotated by homology, nor conserved domain found, and the 9 canonical CPs found, including the one which was automatically annotated as being a LA_52k-L3. Three of the four proteins that clustered with group G1 surface coupling proteins were found to be disordered (>55% disorder), presenting a great percentage of its structure in the form of loops (>50%); more than 48% of their residues exposed, and having less than 5% of intermediate residues, agreeing with G1 protein characteristics [15]. Their isoelectric point, aliphatic index, and the aromatic, positive and negative, residues percentage also fall in the range of G1 proteins, as well as the negative hydropathic index. Most of the characteristics of the two proteins that have clustered with G2 surface coupling proteins also correspond to the characteristics of this group, particularly the degree of disorder and the pI. The high hydropathy index, low disorder, high aliphatic index and high content of aromatic residues and the percentage of loops between 40 and 50% are characteristics of G3 that the two proteins that clustered with this group have. It is a novelty to find CP20k proteins in stalked barnacles, since this group of proteins has been described to be exclusive of acorn barnacles with a calcified base, being located at the interface between cement and the calcareous base, a structure that pedunculate barnacles do not have, and characterized as a calcite-specific coupling protein [11,35,36]. So far, CP20k had never been described in membranous-base barnacles, either pedunculate or membrane-base acorn barnacles, such as *T. japonica* [37].

## 3. Materials and Methods

### 3.1. Sampling, Protein Solubilization, and Extraction

Six immature *Lepas anatifera* (Pedunculata: Scalpellomorpha) specimens (<12 mm in rostro-carinal length) were collected through scuba diving from an oceanographic buoy in Gournes, Crete, Greece, in October 2017. Three replicas composed of 2 individuals each were used for proteomic analysis. Individuals with undeveloped ovaries were selected owing to the proximity of the ovary to the cement gland, to avoid contamination of gland samples with ovary. Animals were brushed to clean epibionts, transported to the laboratory on ice, further swept with an ethanol-soaked cellulose cloth and dissect upon arrival. The cement gland was located according to previous studies [1,2,25]. The tissues collected were kept frozen at −80 °C until protein homogenization and extraction, in SDT buffer (2% SDS, 100 mM Tris/HCl pH 7.6, 0.1 M DTT) according to Campos et al. [42]. Tissues were first homogenized using ultrasounds (Vibra Cell, Sonics, and Materials) at 60 Hz intensity, then mechanically disrupted using microbeads (Precelly’s, Bertin instruments, Montigny-le-Bretonneux, France), followed by incubation in SDT for 14 h with agitation (450 rpm) in a thermomixer at room temperature. Samples were then centrifuged at 16,000× *g* for 20 min, the supernatant collected, the protein concentration determined by spectrophotometry (Synergy HT, BioTek, Winooski, Vermont,) at 280 nm, and stored at −80 °C until further analysis [15].

### 3.2. LC-MS/MS Analyses

Provided lysates were incubated for 30 min with 5 mM tris(2-carboxyethyl)phosphine (TCEP) at 56 °C. The solution was brought to 10 mM TCEP and 10 mM methyl methanethiosulfonate (MMTS) for 15 min, to reduce and protect cysteine residues, respectively [43]. Protein purification, protein digestion, and peptide purification were performed according to a slightly adapted Single-Pot Solid-Phase-enhanced Sample Preparation (SP3) protocol [44,45]. Sequencing grade trypsin (Promega, Fitchburg, WI, USA) was added at a ratio of 1:50 *w*/*w* in 50 mM HEPES, pH 8. After overnight incubation at 37 °C, beads containing the digested peptides were slightly acidified using 10% formic acid (FA), shaken, and incubated overnight at room temperature, after raising the acetonitrile concentration to at least 95%. Adsorbed peptides were washed once with pure acetonitrile (ACN) and then air dried. They were eluted in the first step with 20 µL 2% DMSO for 30 min, and in the second step with 20 µL 0.065% FA, 500 mM KCl in 30% acetonitrile for 30 min. Peptides were vacuum dried and dissolved in 0.2% trifluoroacetic acid/3% ACN for subsequent ultracentrifugation (50,000× *g*, 30 min, RT). LC-MS/MS analyses of purified and desalted peptides were performed on a Dionex UltiMate 3000 n-RSLC system, connected to an Orbitrap Fusion^TM^ Tribrid^TM^ mass spectrometer (Thermo Scientific, Waltham, MA, USA). Peptides of each sample were loaded onto a C18 precolumn (3 μm RP18 beads, Acclaim, 0.075 mm × 20 mm), washed for 3 min at a flow rate of 6 µL/min, and separated on a C18 analytical column (3 mm, Acclaim PepMap RSLC, 0.075 mm × 50 cm, Dionex, Sunnyvale, CA, USA) at a flow rate of 200 nL/min via a linear 120 min gradient from 97% MS buffer A (0.1% FA) to 25% MS buffer B (0.1% FA, 80% ACN), followed by a 30 min gradient from 25% MS buffer B to 62% MS buffer B. The LC system was operated with the Chromeleon software (version 6.8, Dionex, Sunnyvale, CA, USA) embedded in the Xcalibur software suite (version 3.0.63, Thermo Scientific). The effluent was electro-sprayed by a stainless-steel emitter (Thermo Scientific). Using the Xcalibur software, the mass spectrometer was controlled and operated in the “top speed” mode, allowing the automatic selection of as many doubly and triply charged peptides in a 3 s time window as possible, and the subsequent fragmentation of these peptides. Peptide fragmentation was carried out using the higher energy collisional dissociation mode and peptides were measured in the ion trap (HCD/IT).

### 3.3. Protein Identification and Quantitative Proteomic Analyses

MS/MS raw data files comprising three biological replicates per sample studied, gland (DATASET S1) and cement (DATASET S2), were processed independently against *L. anatifera* custom proteins database using MaxQuant freeware (version 1.6.2.3, http://www.maxquant.org). The protein database obtained with TransDecoder (version 5.5.0, https://transdecoder.github.io) comprised 56,606 coding sequences (DATASET S3) from *L. anatifera* transcriptome. From these, 35,405 (64.8% of the total) were annotated using a local BLAST with BLASTp program, against the non-redundant protein database (nr database: ftp://ftp.ncbi.nlm.nih.gov/blast/db; accessed on 1 November 2018), setting a cut-off e-value of 1e^−3^. The DATASETs S1, S2, and S3 were deposited at the Mendeley Data repository with the following Digital Object Identifiers: http://dx.doi.org/10.17632/v2d3hvnycw.1, http://dx.doi.org/10.17632/xztrg72p6f.1, and http://dx.doi.org/10.17632/ff2kwfdjx8.1, respectively. It is noteworthy that this transcriptome was previously published [46] and was obtained from the same specimens used to profile this proteome. The transcriptome is publicly available at Transcriptome Shotgun Assembly (TSA, NCBI), deposited under accession GGJM01000000.

MaxQuant parameters for protein identification were MS and MS/MS tolerances of 20 ppm and 0.5 Da, respectively; two missed tryptic cleavages were allowed; PSMs were accepted at a 1% false discovery rate (FDR) and trypsin was selected for protein cleavage. The modification of cysteine by MMTS (methylthiolation) was set as a fixed modification, while oxidation of methionine and acetylation of protein N-terminus were chosen as variable modifications. Protein quantification was based on approximate absolute protein abundance an intensity Based Absolute Quantification (iBAQ) score calculated by MaxQuant. Venn diagrams were used to identify the shared proteins among the majority proteins of each replicate and figures were built using an online free tool, available at the webserver of the Bioinformatics and Evolutionary Genomics Center (BEG/Van de Peer Lab site, Ghent University, Belgium, http://bioinformatics.psb.ugent.be/webtools/Venn/; accessed date: 23 November 2020).

### 3.4. Data Filtration and Downstream Analyses

Downstream analyses as data filtration of proteinGroups obtained with MaxQuant was performed using Perseus freeware (version 1.6.2.3). Original data filtration included contaminants and REV_removal, as well as those proteins only identified by site. Afterwards, absolute intensity (iBAQ) of filtered proteinGroups was log(x)-transformed and only those proteinGroups with three valid values (of three possible) per row were considered. For the protein expression analyses, only those proteins found in the tree replicates per sample were considered. The resulting matrix containing all filtered proteinGroups was exported and manually reviewed using a set of keywords regarding the family of proteins found in barnacles cement or related organisms. Software used for graphical representation of the results was Excel (Microsoft, Redmond, Washington, DC, USA).

### 3.5. Characterization of Unannotated Cement Proteins

Protein sequences found in the cement proteome without hit or annotations were blasted online against the non-redundant protein database (nr at NCBI), using automatic adjustment of the PSI-BLAST (Position-Specific Iterated BLAST) algorithm. Afterwards, proteins sequences were re-annotated according to hit description, but in most of the cases, no hit was obtained. Proteins were then characterized using ProtParam tool from EXPASY (http://web.expasy.org/protparam/), including molecular weight and isoelectric point, instability index, hydropathy, percentage of positive, negative, and aromatic residues, and aliphatic index. Predictions on the secondary structure composition, solvent accessibility, protein disorder were performed by PredictProtein [47] (https://www.predictprotein.org/) from protein sequences by Meta-Disorder [41]. Principal components analysis (PCA) was used to analyze the relative composition of residues (%) of 19 unannotated proteins identified in *L. anatifera* (Lepadiform Order) cement multicomplex in the present study, plus 9 cement specific proteins (two CP100k, four CP52k, one CP43 and two CP19k) of *L. anatifera* identified and annotated in this work, in comparison to 53 cement specific proteins of various acorn barnacle species (Sessilia Order) and *Pollicipes pollicipes* (Scalpelliform), deposited at NCBI and literature. Only 20 amino acids were considered, for aspartic acid and asparagine were analyzed together, as well as glutamic acid and glutamine since in some cases, CPs’ data delivered by the authors was in this form (one value for each of these two pairs of amino acids. The use of PCA for CPs classification is possible due to the selective pressure observed for the conservation of the relative amino acids composition of these proteins, rather than the conservation of functional domains [15,17], which precludes the possibility of their identification through the homology to others. The higher importance of the relative amino acids abundance over the primary sequence of residues has also been observed in other aquatic invertebrates, namely on the surface coupling proteins of echinoderms [48,49], highlighting the importance of this characteristic on wet adhesion.

## 4. Conclusions

The protein composition of *L. anatifera* cement multicomplex and cement gland was quantitatively studied by the first-time using high-throughput proteomic combined with bioinformatics and statistic approaches. The profiles of both gland and cement proteomes of *L. anatifera* were similar those of the goose barnacle *P. pollicipes*, previously studied. It was dominated by the bulk cohesive proteins CP100k and -52k, whereas surface coupling proteins were less abundant. The species differed on the interfacial proteins, represented in *L. anatifera* mainly by CP43k-like, but also by -19k and -20k, contrarily to *P. pollicipes* adhesive in which only CP19k was found. For the first time CP20k was found to be expressed in a membranous-base barnacle, an interfacial protein postulated to be exclusively related with the adhesion of the cement to the calcareous base of acorn barnacles, which was not the case. Chemical cues were much less represented at *L. anatifera* adhesive as compared to *P. pollicipes*, which we hypothesize having to do with the different reproductive ecology of the species, related to the habitat; one moving as neuston in the oceans, and the other fixed in the rocky shores. Unlike at cement secretion, the canonical barnacle’s CPs could not be detected in the cement gland of *L. anatifera* at the proteomic level, although they did at transcriptomic level. This may have to do with the fact that this species is unable to relocate voluntarily, contrarily to *P. pollicipes*.

Unannotated and uncharacterized proteins accounted for 7.9% of total proteins, of which 6 proteins were listed among the 30 most expressed proteins in the cement proteome of *L. anatifera*. A principal component analyses (PCA) revealed that 8 out of 19 of those proteins were new CPs, since they clustered with the 3 groups of canonical CPs previously described in the literature. Four clustered with surface coupling proteins of the group G1, which includes CP19k, -43k, and -68k; two with the interfacial proteins of G2—the group of CP20k proteins, and two with the proteins of the G3, which comprises CP52k and -100k, the bulk CPs. It remains to be defined if the 11 unannotated CPs that did not cluster with any of the previously defined CP groups have an adhesive or cohesive function, or even a different function in the cement multicomplex. Six of them formed a new cluster together with CP22K and -7K of *A. eburneus*. The importance of PCA on the identification of unannotated non-conserved adhesive proteins, whose selective pressure is on relative amino acids abundance, was demonstrated.

## Figures and Tables

**Table 1:** Characteristics of the nineteen unannotated proteins (upper part of the table) identified in *Lepas anatifera* cement proteome through PCA and nine annotated ones (lower part of the table) using automatic adjustment of the BLASTp program through automatic BLAST against the Non-Redundant protein database (nr at NCBI). No. Res—number of residues; MM—molecular mass; pI—isoelectric point; Neg. res.—negative residues (sum of Asp and Glu); Pos. res.—positive residues (sum of Arg, His, and Lys); Incomplete sequence proteins are presented in italic. In very dark grey are the proteins that have clustered with G2 surface coupling proteins of CP20k kind, in dark grey are those that clustered with bulk proteins (G3), in light grey those that have clustered with G1 of surface coupling proteins, and in very light grey are the proteins that cluster with two previously identified CPs in *Amphibalanus eburneus*: CP7k and -22k. ^¥^—protein misannotated as CP-52k-L3, since as it may be seen by PCA analysis (Figure 6), it clusters with surface coupling proteins (G1), and not with bulk proteins (G3).

iBAQ Rank	Barnacle Specific Leading Cement Proteins	No. Res.	MM (×1000)	pI	Instability Index ^a^	Hydropathy (GRAVY) ^b^	% Neg. Res.	% Pos. Res.	Aliphatic Index ^c^	Aromatic Res. (%)	Secondary Structure Composition (%)			Solvent Accessibility (%)			Protein Disorder ^d^ (%)
											Loop	α-Helix	β-Sheet	Exposed	Interm.	Buried	
4	DN73117_c2_g1_i2.p1	341	39.35	11.37	80.43	−1.603	17.0	24.6	15.43	13.2	96.19	0.00	3.81	91.2	0.59	8.21	70.01
9	DN70620_c0_g3_i1.p2	134	14.93	11.78	34.87	−0.26	4.5	21.6	98.28	5.2	35.82	43.28	20.9	51.49	12.69	35.82	62.69
13	DN70620_c1_g1_i2.p1	163	17.71	9.91	25.09	0.082	6.8	12.9	101.66	4.3	43.56	34.97	21.47	46.63	14.11	39.26	28.22
14	DN65601_c0_g1_i1.p1	538	57.15	5.14	37.24	−0.463	12.4	11.0	71.12	4.4	63.94	9.85	26.21	48.33	5.02	46.65	17.66
23	DN64372_c0_g1_i2.p1	161	17.39	6.29	24.32	0.181	11.8	11.2	107.83	8.7	48.45	23.60	27.95	45.96	6.21	47.83	16.8
26	DN67538_c0_g1_i1.p3	108	12.42	9.05	67.8	−0.936	5.6	13.9	45.41	7.4	72.22	15.74	12.04	64.81	6.48	28.7	81.48
30	DN69760_c0_g1_i1.p1	128	13.38	8.15	41.51	−0.045	9.4	10.2	89.38	3.9	51.59	42.97	5.47	54.69	3.91	41.41	78.9
42	DN61611_c0_g1_i1.p1	413	42.31	6.07	35.18	−0.289	9.7	7.8	71.67	2.2	79.18	11.62	9.2	61.5	3.39	35.11	55.2
44	DN63562_c0_g1_i1.p1	420	43.24	10.18	66.90	−0.979	10.9	15.0	42.71	4.7	97.86	0.00	2.14	92.62	1.19	6.19	97.6
46	DN64031_c0_g1_i1.p1	368	40.99	9.75	60.52	−0.791	12.0	15.5	67.04	4.9	71.47	12.50	16.03	56.52	2.72	40.76	30.16
52	DN61926_c0_g1_i3.p1	112	12.10	9.25	30.56	0.004	4.5	16.1	68.04	8.1	66.07	25.00	8.93	52.68	5.36	41.96	60.71
53	DN56671_c0_g2_i1.p1	116	12.63	6.26	44.18	0.199	9.5	8.6	95.95	6.9	49.14	44.83	6.03	49.14	6.03	44.83	47.41
54	DN72668_c0_g1_i2.p1	1120	124.85	9.27	49.24	−0.584	10.5	14.1	66.96	7.4	63.48	17.32	19.2	51.07	5.09	43.84	28.75
61	DN69827_c0_g1_i5.p1	285	30.58	5.39	81.17	−0.672	11.9	9.5	57.19	6.4	94.74	2.81	2.46	77.89	2.81	19.3	90.18
66	DN53050_c0_g1_i2.p1	112	12.56	5.26	43.07	−0.848	17.9	16.1	52.95	8.1	66.96	14.29	18.75	71.43	6.25	22.32	97.54
89	DN67416_c2_g1_i13.p1	122	12.47	9.93	42.42	−0.301	8.2	9.8	70.33	4.9	66.39	22.13	11.48	68.03	2.46	29.51	77.87
93	DN62666_c1_g1_i3.p1	312	34.10	5.46	59.45	−0.508	13.5	10.9	84.1	3.5	75.32	12.50	12.18	56.41	3.85	39.74	17.63
116	DN62022_c1_g1_i2.p1	344	37.47	10.03	48.67	−0.394	9.6	16.6	83.63	4.4	57.56	22.67	19.77	49.13	9.59	41.28	23.84
118	DN58739_c0_g1_i1.p1	171	18.71	9.38	75.75	−0.309	7.0	14.6	76.9	4.6	76.61	10.53	12.87	52.05	8.19	39.77	87.72
1	DN69987_c0_g1_i2.p1 (LA100k)	924	99.8	9.81	10.07	0.205	5.8	8.1	107.6	8.60	54.11	35.61	10.28	37.01	9.52	53.46	0.32
2	DN63945_c0_g1_i4.p1 (LA52k)	380	43.3	10.38	50.11	−0.104	2.4	12.1	89.6	15.8	55.79	42.37	1.84	38.84	12.37	50.79	0.8
3	DN40455_c0_g1_i1.p1 (LA43k-L)	484	46.9	4.42	38.05	−0.323	8.9	5.4	67.9	1.40	92.98	3.10	3.93	91.32	0.62	8.06	90.70
5	DN67731_c0_g1_i1.p1 (LA52k)	775	85.6	10.06	41.77	0.033	3.0	10.1	101.5	12.70	65.55	26.06	8.39	35.23	11.61	53.16	0.00
6	DN69987_c0_g2_i1.p1 (LA100k)	155	16.5	6.08	39.81	0.228	6.5	5.8	113.3	5.70	57.42	40.00	2.58	60.00	4.52	35.48	0.0
8	DN63945_c0_g1_i2.p1 (LA52k)	380	43.2	10.43	49.74	−0.113	2.4	11.8	89.4	15.60	56.32	41.84	1.84	36.84	12.11	51.05	2.37
21	DN62610_c0_g1_i4.p1 (LA52k)	1253	134.1	10.11	54.20	0.126	5.9	9.3	102.7	6.10	73.18	22.91	3.91	52.19	6.70	41.10	29.93
31	DN66462_c0_g1_i1.p1 (LA52k-L3) ^¥^	267	26.7	4.58	30.19	−0.061	12.4	7.5	83.5	2.20	73.78	16.85	9.36	73.03	0.37	26.59	83.52
33	DN68159_c0_g1_i3.p1 (LA19k)	218	21.7	4.50	50.61	−0.172	13.3	6.9	78.0	1.80	68.81	6.88	24.31	68.81	3.21	27.98	88.07

^a^ Instability index (II)—provides an estimate of the stability of the protein in a test tube, depending on the presence of certain dipeptides [38], the occurrence of which is significantly different in the unstable proteins compared with those in the stable ones. A protein whose instability index is smaller than 40 is predicted as stable, a value above 40 predicts that the protein may be unstable. ^b^ GRAVY—Grand Average of Hydropathy—The GRAVY value for a peptide or protein is calculated as the sum of hydropathy values [39] of all the amino acids, divided by the number of residues in the sequence. Values define relative hydrophobicity of amino acid residues, the more positive the value, the more hydrophobic in the amino acids located in that region of the protein. ^c^ Aliphatic index of a protein is defined as the relative volume occupied by aliphatic side chains (alanine, valine, isoleucine, and leucine). It may be regarded as a positive factor for the increase of thermostability of globular proteins [40]. ^d^ Protein disorder—percentage of disordered regions as compared to the total protein sequence length predicted by Meta-Disorder [41].

## Data Availability

Data are publicly available at Mendeley Data repository http://dx.doi.org/10.17632/v2d3hvnycw.1; http://dx.doi.org/10.17632/xztrg72p6f.1; and http://dx.doi.org/10.17632/ff2kwfdjx8.1.

## Supplementary Materials

Supplementary materials can be found at https://www.mdpi.com/1422-0067/22/7/3370/s1.
